# The impediments of implementing infection prevention control in public hospitals: Nurses’ perspectives

**DOI:** 10.4102/hsag.v27i0.2033

**Published:** 2022-11-11

**Authors:** Thizwilondi A. Magadze, Tinyiko E. Nkhwashu, Sophy M. Moloko, Dayanithee Chetty

**Affiliations:** 1 Department of Nursing, Faculty of Health Science, Sefako Makgatho Health Sciences University, Ga-Rankuwa/Pretoria, South Africa; 2 Department of Health, Faculty of Nursing, Ann Latsky Nursing Campus, Johannesburg, South Africa; 3 Department of Nursing, Faculty of Health Science, Sefako Makgatho Health Sciences University, Pretoria, South Africa

**Keywords:** impediments, infection prevention and control measures, nurses, implementing IPC measures, public hospitals

## Abstract

**Background:**

Infection prevention and control (IPC) programmes were introduced to combat healthcare-associated infections and antimicrobial resistance and to facilitate the implementation of IPC measures. The implementation of policies and guidelines results in effective service delivery.

**Aim:**

The purpose of the study was to explore nurses’ experiences and perceptions regarding the impediments to implementing the IPC measures.

**Setting:**

The study was conducted in three public hospitals in Gauteng Province, South Africa.

**Methods:**

A qualitative exploratory design was used, and 49 nurses were purposively selected from three public hospitals to understand their experiences and perceptions regarding the impediments to implementing the IPC measures. Five focus group discussions were conducted using semi-structured interviews. Tesch’s eight steps method was used to analyse data resulting in three main themes and seven sub-themes.

**Results:**

Three main themes emerged. Nurses experienced challenges regarding knowledge and attitudes towards IPC measures, inadequate hospital infrastructure and lack of management support.

**Conclusion:**

The lack of infection prevention control knowledge, infrastructure and management support impede the implementation of IPC measures. Consistent support in terms of training, resources and infrastructure is essential for implementing the measures.

**Contribution:**

The findings in this study will empower the nurses, doctors and managers with knowledge in implementing IPC measures to improve the infection prevention programme.

## Introduction

Infection prevention and control (IPC) is a scientific evidence-based approach and feasible solution designed to prevent harm caused by infection to healthcare users and workers (National Department of Health 2020a, 2020b). Preventing harm to health workers, patients and visitors because of infection in healthcare facilities is fundamental to achieving quality care, patient safety, health security and the reduction of healthcare-associated infections (HAIs) and antimicrobial resistance (AMR). The prevention of harm can only be achieved by implementing the IPC programme with clear policies, guidelines and measures. The IPC measures include standard precaution (SP), transmission-based precautions, building environment, material and equipment for IPC, surveillance of HAIs, antimicrobial stewardship, outbreak response, reporting of notifiable medical conditions and education and training of staff and monitoring and evaluation (National Department of Health 2020a).

The effective implementation of the IPC measures requires the use of multimodal strategies (MMSs), which involve the engagement of various stakeholders with clear allocated responsibilities to ensure commitment and sustainability. The World Health Organization (WHO) identified elements of MMS towards ensuring that the IPC is practised throughout the health system as follows: (1) availability of the appropriate infrastructure and supplies to enable the implementation of IPC, (2) education and training of health workers and key role-players, (3) monitoring the infrastructure, practices, processes, outcomes and providing feedback, (4) reminders and communication of improvements in the workplace and (5) culture change within the health facility or the strengthening of a safety climate (National Department of Health 2020a & 2020b; WHO 2016).

Despite the availability of the guidelines on IPC measures, the National Department of Health (2020) and WHO (2016) found that healthcare workers were not implementing the IPC measures appropriately. A 2017 Joint External Evaluation (JEE) of International Health Regulations (IHR) Core Capacities of the Republic of South Africa (RSA) report shows that the country obtained a low score of one (no capacity) out of five (sustainable capacity) on the indicator for HAIs and IPC, meaning that the country does not have enough capacity to prevent HAIs (National Department of Health 2020a).

According to Henderson et al. (2021) and Houghton et al. (2020), several factors were found to impact the implementation of the measures. The factors are classified into systematic, organisational, environmental and individual factors. Systematic factors include material and human resources issues and policies that affect the implementation of infection control measures. The organisational factors relate to managerial style and support, interprofessional relationships and budgetary factors. Environmental factors relate to features of the physical layout of the hospital, availability of isolation rooms and hand-wash basins. The individual or personal factors relate to the knowledge, attitudes and beliefs of the individual about infection control. Some healthcare workers were not wearing personal protective equipment (PPE) when dealing with suspected infectious patients, while some were using the same PPE (gowns and masks) with all patients (El Bushra et al. 2017). The lack of sufficient PPE, high workload and lack of management support on IPC were identified as the common causes of not implementing the IPC measures (Henderson et al. 2021). Schmidt et al. (2020) attest that inadequate IPC in hospitals has been shown to have several consequences. These include increased bed occupancy and a strain on drugs and other scarce hospital resources, and AMR, thus lengthening the duration of patient stay in the hospital. Furthermore, this translates into high costs for the hospital and the patient and increased social suffering for the patient and family.

While other healthcare workers (including doctors, physiotherapists and occupational health therapists) are at risk of transmitting and acquiring HAIs, the study focused on nurses because they are at higher risk of contracting and transmitting HAIs if they do not implement the IPC measures. The nurses spend most of their time with patients as compared with other healthcare workers, and they form the most significant cadre of health workers (Barrera-Cancedda et al. 2019). They are, therefore, expected to lead the IPC programme and practise the measures effectively. Hence, this study aimed to explore nurses’ experiences and perceptions regarding the impediments to implementing the IPC measures at the hospitals in Tshwane District, Gauteng Province, South Africa. The study is imperative in developing strategies and guidelines to promote the implementation of IPC measures.

## Research methods and design

### Study design

This study was part of a mixed-method study designed to develop guidelines to improve the implementation of IPC measures in public hospitals in Gauteng Province. The article reports on a qualitative strand, which explores nurses’ experiences and perceptions regarding the impediments to implementing the IPC measures. A qualitative exploratory-descriptive design was deemed appropriate for exploring the impediments from the people living the experiences, the nurses (Gray & Grove 2021).

### Study setting

The study was conducted in three public hospitals in the Tshwane District, Gauteng Province, South Africa. The hospitals are situated in the north and east part of the Tshwane District. The three hospitals are about 49 km apart. The hospitals were selected based on their differences in their hierarchical level of care. One is a district hospital, one is a regional hospital, and the other one is an academic hospital.

### Population and sample

The study population comprised of registered, and enrolled nursing assistants working directly with patients in the wards. The nurses were purposefully selected because of their experience implementing IPC measures as part of their routine nursing care. Forty-nine nurses participated in five focus group (FG) discussions. Four FGs consisted of 10 members, and one had nine members. Two FGs were conducted in the district hospital, one in the regional hospital and two in the academic hospital.

### Recruitment and data collection

Detailed letters about the study were sent to nursing managers to request meetings with nurses after permission to conduct the study was obtained from the chief executive officers of the respective hospitals. Participants were recruited through several meetings held with the respective nurses. Appointments for FG discussions were arranged with nurses who agreed to take part in the study. The FG discussions took place in nursing managers’ boardrooms in the respective hospital. The nursing managers’ board rooms provided privacy and were free from interruptions.

An English semi-structured self-developed interview guide was used to collect data during the FG discussions. The discussions were focused on nurses’ experiences and perceptions about factors impeding the implementation of the IPC measures in the selected hospitals. Further questions were on possible interventions required to improve the implementation of the IPC measures. The researcher facilitated and managed the discussions. Follow-up questions and probes were used in response to the responses from the participants. An audio-recorder was used to record the discussions per the participants’ consent to maintain the data accuracy. Field notes were recorded (Polgar & Thomas 2020). The duration of each FG ranged from 45 to 60 min. Data saturation was reached when no new information was discovered (Labiondo-Wood & Haber 2018).

### Data analysis

Recorded data and field notes were transcribed verbatim. The data were analysed using Tesch’s method, following the eight steps as outlined in Creswell (2020). The data were analysed as described below.

The researcher carefully read through all the data collected to better understand the information contained therein and identify new ideas in the data. A list of all the topics identified from the data topics was prepared. The topics were compared and similar topics grouped, which enabled the coding of significant themes that emerged. The codes were written next to the appropriate segments of the text from which the information was derived. The categories were reduced by combining coded information that had some similarities. A verification was carried out to remove duplications in categories and alphabetical abbreviations. Finally, information belonging to each category was grouped, and a preliminary analysis was performed.

### Measures of trustworthiness

Four criteria of trustworthiness guided by Lincoln, Lynham and Guba (2018) were adopted and operationalised, namely credibility, transferability, dependability and conformability. To ensure credibility, a trusting relationship was built with the participants to familiarise them with the researchers and the environment. Frequent and relevant follow-up probing questions enhanced participants’ engagement. The researchers depended on field notes and an audio-recorder when transcribing data to ensure that no information was missed. Transferability was ensured by the researchers describing the study design and methods. To ensure confirmability, the researchers used bracketing to ensure that preconceived ideas and biases did not influence the results. Reflexivity was applied by creating a reflexive diary to record and reflect on thoughts and previous experiences about the phenomenon. The reflections enabled the researchers to probe and understand the nurses’ experiences and perceptions regarding the impediments to implementing IPC measures.

### Ethical considerations

Ethical approval was sought from the Sefako Makgatho Health Sciences University Research Ethics committee (SMUREC/H/228/2018: PG). Permission to conduct the research was obtained from the different health authorities: the chief executive officers and the clinical directors of the hospitals to ensure ethical compliance of the research and approval at the hospitals. Ethical research principles of the right to self-determination, right to privacy, right to anonymity and confidentiality, right to fair treatment and right to protection from discomfort and harm were adhered to. Informed consent was obtained from each participant before undertaking the research.

## Results

### Demographic characteristics of the participants

Forty-nine nurses, including 13 males and 36 females, participated in five FGs. Among the five FGs, all but one had 10 members, while the other had nine members. Focus group 1 from the district hospital consisted of five registered nurses, four enrolled nurses and one enrolled auxiliary, FG2 had four registered nurses, two enrolled nurses and four enrolled auxiliary nurses, FG3 from the regional hospital consisted of five registered nurses, two enrolled nurses and three enrolled auxiliary nurses, FG4 from the academic hospital had five registered nurses and four enrolled nurses; while, FG4 had five registered nurses, four enrolled nurses and one enrolled auxiliary nurse. Fourteen registered nurses had nursing diplomas, nine had bachelor’s degrees, and one had a master’s degree. Sixteen enrolled nurses and nine enrolled auxiliary nurses held enrolled nursing certificates and enrolled auxiliary nursing certificates, respectively. Table 1 displays the characteristics of the study participants.

**TABLE 1:** Participant characteristics.

Variables	Focus group 1: District hospital (*n* = 10)	Focus group 2: District hospital (*n* = 10)	Focus group 3: Regional hospital (*n* = 10)	Focus group 4: Academic hospital (*n* = 9)	Focus group 5: Academic hospital (*n* = 10)
**Gender**					
Male	7	1	4	0	1
Female	3	9	6	9	9
**Age**					
18–30	4	6	2	2	3
31–40	2	2	1	3	2
41–50	3	1	1	3	3
51–65	1	1	6	1	2
**Categories of nurses**					
Registered nurses	5	4	5	5	5
Enrolled nurses	4	2	2	4	4
Enrolled nursing auxiliary	1	4	3	0	1
**Nursing qualification obtained**					
Master’s degree	0	0	0	1	0
Bachelor’s degree	2	2	2	2	1
Diploma in nursing	2	3	2	3	4
Certificate in nursing	5	5	6	3	5
**Area of work**					
Surgical ward	5	3	5	1	8
Medical wards	2	5	4	7	0
ICU	2	1	0	1	1
Theatre	1	1	1	0	1
**Work experience**					
0–5 years	2	2	2	2	2
6–10 years	7	7	7	5	7
11 years and above	1	1	1	2	1

### Experience about Quality improvement plans (QIPs) related to infection prevention and control measures

Out of 49 participants, only 17 have been involved in quality and ward infection control forums. All 49 nurses’ participants have knowledge of IPC. Participants were knowledgeable regarding their roles and responsibilities concerning patient care, quality improvement and their duty to report concerns. Participants confirmed that there is an IPC policy and procedures which were embedded into practice in most of their wards.

## Themes and sub-themes

Three themes with related sub-themes emerged from the data analysis, as shown in Table 2.

**TABLE 2:** Themes and sub-themes emerging from the study.

Themes	Sub-themes
1. Challenges regarding knowledge and attitudes towards infection prevention and control measures	1.1. Knowledge and attitudes of nurses and doctors 1.2. Knowledge and attitudes of management
2. Inadequate hospital infrastructure	2.1. Poor hospital maintenance 2.2. Structural and capacity challenges
3. Lack of management support	3.1. Inadequate resources 3.2. Poor communication and lack of support 3.3. Inappropriate education and training Measures

### Theme 1: Challenges regarding knowledge and attitudes towards infection prevention and control measures

The participants expressed challenges regarding the knowledge and attitudes of healthcare providers and managers towards IPC measures.

#### Sub-theme 1.1: Knowledge and attitudes of nurses and doctors

Participants indicated that some nurses and doctors lack IPC knowledge, and some display negative attitudes towards implementing IPC measures even after the in-service training. They do not implement the IPC measures, and some doctors do not appreciate advice from nurses. The practice exposes other nurses to medico-legal hazards.

‘Some nurses [*scratching the head*] does not know a thing. They must be re-trained if the training was ever done.’ (FG5, P8)

‘This staff nurses do wrong wound dressing even after we train them several Times.’ (FG3, P6)

‘There is too much resistance when it comes to infection control-related issues, and it is just attitude.’ (FG1, P2)

‘Some doctors and nurses throw the waste everywhere, and if you try to speak, they give attitude.’ (FG2, P9)

To improve the knowledge and attitudes, the participants recommended that healthcare staff be orientated on IPC measures. They should work collaboratively as a team to support each other.

‘Every personnel working near-patient need to be orientated on IPC.’ (FG1, P3)

‘For a better chance of implementing IPC issues, nurses must support each other and work as a team, while we also change our attitude.’ (FG4, P6)

#### Sub-theme 1.2: Knowledge and attitude of nursing management

Some participants expressed that the managers do not have knowledge and insight into implementing IPC measures. The nurses felt poorly treated, and the managers did not recognise their challenges.

‘The management want us to comply with IPC measures that they themselves are not adhering to.’ (FG3, P1)

‘Nursing managers does not even know the infection control issues. That is why they do not care to understand shortages.’ (FG3, P5)

‘Nursing service managers visit the wards to bother us, and they do not consider our challenges about IPC issues.’ (FG2, P7)

Participants suggested that managers must be trained to improve their knowledge and insight into IPC measures.

‘Management must have adequate knowledge of information available in the IPC policy; they must be trained like us.’ (FG4, P2)

### Theme 2: Inadequate hospital infrastructure

Participants highlighted challenges with the condition of the infrastructure at the hospitals. The infrastructure was not adequate to allow effective implementation of the IPC measures.

#### Sub-theme 2.1: Poor hospital maintenance

The participants mentioned that some of the essential infrastructures required for implementing the IPC were not working, mainly the hand-washing basins and the toilets, while some of the areas in the hospital did not have lighting, and the ceiling was damaged.

‘Most of the hand-washing basins taps are not working, and toilets are leaking.’ (FG4, P7)

‘In other areas of the ward, ceiling has fallen, lights are not working.’ (FG2, P9)

Some participants further indicated that pests were all over the place, which hindered the implementation of the infection prevention programme. They suggested that the hospitals must hire skilled people to do the fumigation.

‘Even though they say they are fumigating, there are lots of cockroaches….’ (FG1, P1)

‘I think they must work or hire people who know this work.’ (FG1, P10)

#### Sub-theme 2.2: Structural and capacity challenges

Participants articulated the hospital structure and capacity as factors affecting the implementation of IPC measures. There are no isolation areas, patients are placed in corridors, and babies are sharing beds, thus increasing the transmission of infections.

‘We do not even have isolation facilities.’ (FG2, P8)

‘So, if the ward is overcrowded, patients are put in the corridor and obstruct hand washing basins, how are we going to wash our hands.’ (FG1, P4)

‘Due to shortage of space, many babies are sharing cubicles in our wards.’ (FG5, P5)

### Theme 3: Ineffective management practices

Participants felt that ineffective management practices in their working environment affect the implementation of IPC measures in the hospitals. These are inadequate material and human resources, poor communication and support, and a lack of orientation and training on IPC measures.

#### Sub-theme 3.1: Inadequate material and human resources

Participants expressed a lack of management support regarding resources required for implementing the IPC measures. They indicated that they have a shortage of PPE, hand-washing soap, alcohol hand rub and hand paper towels, which are critical to minimise the risk of infection transmission.

‘This hospital management does not care, and there is nothing, I mean no resources for IPC in the institution.’ (FG3, P9)

‘Supply chain always tells us that they do not have material resources related to IPC such as alcohol hand rub, gloves, gowns and other PPE. We are expected to wear aprons as protective clothing, but it is not available.’ (FG1, P9)

‘There are no masks, and I have a patient with TB in that ward.’ (FG2, P5)

To improve the supply of resources, participants recommended that healthcare management must work with supply chain management and ensure that the supply chain is held accountable for shortages of resources.

‘Management must be involved in issues of the supply chain.’ (FG4, P7)

‘Supply chain must be held accountable for the stock that is always not there. This puts us at risk.’ (FG2, P9)

The lack of human resources was highlighted as a hindrance to the effective implementation of the IPC measures. Some participants could not leave the ward to attend to IPC capacity measures because of staff shortage.

‘It is difficult to implement some measures when we are short staffing.’ (FG5, P2)

‘I cannot attend workshops and meetings of IPC due to shortage in the ward.’ (FG3, P7)

Participants recommended the recruitment of more nurses to improve the implementation of IPC measures.

‘Can’t they see we need more hands (hands raised), they must employ more nurses to assist.’ (FG5, P9)

#### Sub-theme 3.2: Poor communication and support

Participants expressed a lack of communication between management and the staff members. They also perceived that management does not support them adequately and is very reactive when approaching IPC situations instead of being proactive. The lack of communication and reactive practice affect the implementation of IPC measures.

‘The other issue might be the way we communicate. We get important information by surprise and this frustrated us.’ (FG1, P10)

‘Like we are not properly informed on what is happening about infection prevention and control policies….’ (FG4, P9)

‘Our managers are very reactive. They come only when there is a bad situation, an infection control incident. They do not come when we have many patients to see what has to be done or how are we coping regarding IPC measures.’ (FG1, P9)

To improve communication and support from management, participants suggested that management should participate in their IPC meetings to gain insight into the IPC challenges and plans.

‘I wish management listen to us when we have challenges related to infection prevention and control.’ (FG2, P10)

‘Management must participate and be involved with us when planning all infection control issues.’ (FG4, P1)

#### Sub-theme 3.3: Lack of orientation and training on infection prevention and control measures

Some participants articulated a lack of training and orientation on IPC measures among nurses as challenges to effective implantation of IPC measures.

‘Yoh, I work in this ward; these nurses do not know what to do as they did not get the in-service training on infection prevention and training.’ (FG2, P1)

‘I think most of our nurses never get in-service training on IPC issues.’ (FG4, P4)

Participants acknowledge that in-service training will contribute a lot to implementing IPC measures. To improve the application of the IPC knowledge, the participants suggested that the training should be started with the students at the respective higher education institutions.

‘If all of our nurses are in-serviced on IPC, which will help a lot.’ (FG3, P9)

‘But, because we are getting training from schools, the knowledge will be beneficial to us than be taught many policies as new during employment. I agree that we can perform better than this if the training was offered at that time.’ (FG1, P7)

## Discussion

The findings of this study show that some healthcare workers do not implement IPC measures because of a lack of knowledge. The lack of knowledge and skills on IPC measures results in nurses perceiving IPC as impossible to implement, thus developing negative attitudes towards the IPC (Singh, Wiese & Sillerud 2019). Training is considered necessary in improving the knowledge and skills to implement the IPC measure and manage the challenges, thus improving nurses’ interest and attitudes towards the IPC (Desta et al. 2018). However, the study findings show that training alone is insufficient to improve the implementation of the IPC measures, as even the nurses who acquired knowledge through the in-service training do not implement the IPC measures. Similarly, a study in North-Western Nigeria found low implementation of the IPC measures among the nurses, despite the knowledge they acquired through training (Iliyasu et al. 2016). Therefore, knowing IPC measures and a positive attitude do not ensure adherence to the policies related to IPC (Refeai et al. 2020).

In addition to the nurses, it appears that nursing managers in the hospitals lack insight into the implementation of IPC measures; hence they fail to recognise the challenges affecting the execution. Instead of supporting the nurses, they develop negative attitudes towards them. The attitudes of the managers cause frustration among the nurses and create an unfavourable working environment and non-adherence to IPC measures (Schwappach 2018). Non-adherence to IPC measures affects the provision of quality services and contributes to infections and disease outbreaks (Refeai et al. 2020).

The study shows poor interpersonal relationships between managers and nurses, which is unhealthy and considered detrimental to the success of the IPC programme (Amukugo, Nangombe & Karera 2020). The managers’ attitudes and behaviours are reflected by nurses who regard them as role models and are influential in driving or limiting the nurses to accomplish the goals of the IPC programme (Schwappach 2018). Hence, a good relationship between nursing managers and other healthcare workers is considered a prerequisite for employees to be enthusiastic and actively involved and committed to implementing the IPC measures (Qalehsari, Khaghanizadeh & Ebadi 2017).

The poor maintenance of the infrastructure in the hospitals hinders the implementation of the IPC measures. Hand washing basins are critical in implementing the IPC measures (Manchanda, Suman & Singh 2018). However, this study found that nurses did not always practise hand washing because some of the hand-washing taps are damaged, leaving them with a few that are not always close to the patients. Similar structural issues were found to be a concern in neonatal units in the Western Cape Province, South Africa, whereby hand-washing basins were far from the patient care areas. Healthcare workers had to improvise hand hygiene measures that were also seen as a risk for infection control (Olivier et al. 2018).

The presence of pests in hospitals is not unique to this study. Studies conducted in Algeria, Ghana and Libya found pest infestation common in many hospitals, mainly cockroaches. The presence of pests in healthcare settings exposes the personnel and patients to infections and reflects poor hospital maintenance (Donkor 2019; Garboui & Elbagrmi 2017; Menasria et al. 2014). Donkor (2019) asserts that pests harbour and disseminate microbial pathogens and are reservoirs for antimicrobial-resistant organisms. They are involved in the transfer of nosocomial infections in the hospital’s environment, consequently, increasing the rate of HAIs and AMR among patients. To reduce the rate of HAIs and AMR, the literature suggests the inclusion of pest control measures and hospital infrastructural maintenance in IPC strategies (Donkor 2019; Garboui & Elbagrmi 2017).

Overcrowding because of lack of space and bed capacity in this study made separating infectious and non-infectious patients impossible, thus compromising IPC measures. Manchanda et al. (2018) assert that IPC measures are always compromised during overcrowding. Furthermore, sharing rooms and bathroom facilities exposes patients to cross infections (Henderson et al. 2021). The lack of isolation facilities reported in this study violates the implementation of the National Infection Prevention and Strategic Framework (Houghton et al. 2020; National Department of Health 2020), which regards isolation rooms as a necessity for controlling the spread of infections. A healthcare facility environment with sufficient space to isolate patients is vital for managing and controlling cross infections (National Department of Health 2020a).

The practice of having babies sharing cubicles because of shortages is not unique to this study. A study on preventing and treating nosocomial infections found that neonates are vulnerable to sharing beds because of a shortage of material resources and infrastructure. The sharing increases the risk of cross-infection among neonates (Ramasethu 2017). To prevent cross-infection, the hospital infrastructure should be well maintained. Keeping the hospital building repaired and environmentally user-friendly enhances excellence in clinical practice (Chabrol, Albert & Ridde 2019).

The hospitals in this study have a shortage of material and human resources required for implementing the IPC measures. The shortages include the essential resources for IPC, like hand-washing soap, alcohol hand rubs, gloves and other PPE. The shortage of resources is negatively influencing the implementation of IPC measures in health facilities (Henderson et al. 2021; Vukoja, Riviello & Schultz 2018). The cause of the shortage in this study appeared to be a lack of knowledge and understanding of essential resources for IPC by the supply chain unit. Therefore, it is recommended that the healthcare providers be involved in the procurement process by identifying IPC resources that are routinely used when providing care. The involvement of healthcare providers is envisaged to improve the supply of resources and limit overstocking unnecessary ones, consequently preventing the purchasing of poor quality PPE (Vukoja et al. 2018).

Furthermore, the study found a shortage of staff to be a challenge in implementing the IPC measures. The impact of staff shortage on adherence to IPC was also reported in a study conducted in New York, where nurses found it difficult to comply with IPC guidelines because of the high workload (Pogorzelska-Maziarz et al. 2020). Non-compliance to IPC guidelines increases the risk of HAIs and AMR among patients and nurses. As a means of strengthening the implementation of the IPC programme, it is, therefore, necessary to ensure adequate staffing (Alsuhaibani et al. 2022). Hence, one of the duties of nursing management is to ensure that there are sufficient personnel to provide quality care (Griffiths et al. 2020).

Poor communication and lack of support from management are perceived as hindrances to implementing the IPC measures in this study. It appears that there are no clear IPC communication channels in this study; hence, the nurses complain that they are not adequately informed about the IPC policies. The lack of clear communication channels and ambiguous messages from management causes frustration in employees, consequently affecting the implementation of the IPC measures (Al Shamsi et al. 2020). Clear communication is critical in improving the IPC implementation process. Through communication, nurses can recognise the challenges in their behaviour and set goals to improve this process (Jones, Vaux & Olsson-Brown 2019). Nurses in the current study perceive managers as reactive and fault-finding because they only engage nurses on IPC measures when adverse incidents are reported on IPC. Managers do not conduct IPC support visits regularly. The lack of support from managers is seen as a key contributor to employee disengagement in the workplace, whereby the employee feels excluded from the organisation’s decision-making and planning process (Al Shamsi et al. 2020). In contrast, the practice of managers making rounds in the wards and offering words of encouragement is perceives as supportive of staff and promotes their engagement with IPC (Houghton et al. 2020).

Nurses in the study acknowledge the negative impact of a lack of training on implementing IPC measures among nurses and managers. Lack of training about the specific infection control measures and the use of PPE contribute to poor implementation of IPC measures (Houghton et al. 2020).

Although they regard nursing managers as responsible for ensuring that healthcare workers undergo training, it is vital that nursing managers also undergo training on IPC measures. The training will ensure that the nurses, doctors and managers understand IPC measures (Price & Reichert 2017). Managers will develop an insight into IPC measures, thus enabling them to provide the necessary support to the nurses and doctors so that they could have the same understanding of implementing infection prevention. Therefore, training in the form of continuous professional development is an expectation and a need for nurses across their careers. The training will assist in developing nurses’ and doctors’ abilities to provide quality patient care (Price & Reichert 2017).

## Conclusion

The study showed that a lack of IPC knowledge, communication, dedication and management support impedes the implementation of IPC measures. Poor interpersonal relations between managers and staff result in staff developing negative attitudes towards the managers and the IPC programme. Poor infrastructures like limited hand-washing stations, isolation facilities, poor pest control and overcrowding compromise the implementation of IPC measures and increase the risk of spreading infections. The lack of PPE, hand-washing soap and alcohol rub reflects poor management support for the IPC programme.

The findings suggest that nurses understand the importance of implementing IPC measures and recognise the need to rectify the impediments. Hence, the empowerment of the nurses, doctors and nursing managers with knowledge and skills needs to take place. The empowerment through training will bring good working relationships and effective communication among the healthcare workers because everyone would have insight into IPC and be aware of what is expected of them regarding the implementation of IPC measures. The Department of Health should develop an incentive system to acknowledge and motivate the healthcare providers in implementing the IPC measures. The incentive might improve the nurses’ morale and make them feel they have support from the nursing management. Adequate staff, sufficient material resources and infrastructure are necessary for successful implementation of the IPC programme. Further research should be undertaken to measure the impact of training healthcare workers on IPC measures. The evaluation should focus on healthcare workers’ satisfaction with the training and the relevance in improving the implementation of the measure.

### Limitations

The study results cannot be generalised to other settings but may be transferable to hospitals with similar contexts. The researchers provided a detailed description of the study’s design, context and participants to allow the reader to draw conclusions about the translation of the results to other settings. The study was conducted on three different levels (district, regional and academic hospitals); however, the themes were not classified per hospital because they were similar.
